# Relationship between perfectionism and attitudes toward doping in young athletes: the mediating role of autonomous and controlled motivation

**DOI:** 10.1186/s13011-020-00259-5

**Published:** 2020-02-27

**Authors:** Kun Wang, Lei Xu, Jimeng Zhang, Dong Wang, Kaihong Sun

**Affiliations:** 1 Department of Physical Education, Shanghai Jiao Tong University, Shanghai, China; 2 School of Physical Education, Yangzhou Polytechnic University, Yangzhou, Jiangsu China

**Keywords:** Athletes, Perfectionism, Autonomous motivation, Controlled motivation, Attitudes toward doping

## Abstract

**Background:**

Recent studies suggest that autonomous and controlled motivation may mediate the relationship between perfectionism and attitudes toward doping. The purpose of this study was to investigate the association among perfectionistic strivings, perfectionistic concerns, autonomous motivation, controlled motivation, and attitudes toward doping in young athletes. The potential mediating role of autonomous and controlled motivation was examined.

**Methods:**

Two hundred and forty-three Chinese athletes were surveyed using questionnaires. Structural equation modeling (SEM) was conducted to evaluate the potential mediating role of autonomous and controlled motivation between perfectionism and attitudes toward doping.

**Results:**

The results showed that perfectionistic strivings was negatively associated with attitudes toward doping, whereas perfectionistic concerns positively predicted attitudes toward doping. Autonomous motivation was negatively associated with attitudes toward doping, whereas controlled motivation positively predicted attitudes toward doping. The mediation analyses showed that the relationship between perfectionistic strivings and attitudes toward doping was partially mediated by autonomous motivation. The relationship between perfectionistic concerns and attitudes toward doping was partially mediated by controlled motivation.

**Conclusions:**

The findings suggested that fostering perfectionistic strivings and autonomous motivation and reducing perfectionistic concerns and controlled motivation may be considered for inclusion in athletes’ anti-doping education.

## Introduction

Doping not only ethically violates the principle of fair competition in the Olympics, but also seriously impairs athletes’ physical health. Therefore, the International Olympic Committee has sounded the clarion call of anti-doping since 1968 and continues to increase the testing and punishment of doping. Nevertheless, the issue of athletes’ use of doping has not yet been completely resolved and doping scandals are still frequently reported. Obviously, in the anti-doping effort, it is not sufficient to rely only on rigorous checks and crackdowns. It is suggested that changing athletes’ attitudes toward doping may be an important strategy for dealing with the problem of repeated use of doping by athletes [1]. Currently, exploring the potential antecedents that can effectively predict and influence athletes’ attitudes toward doping has become a hot topic in sport psychology research [2, 3].

Among the personality traits that affect the attitudes toward doping, some scholars are concerned about the perfectionism tendency of athletes [4–6]. Perfectionism is often defined as a type of personality that seeks perfection and sets excessive standards, along with a tendency to over-evaluate one’s behavior [7]. Studies have shown that perfectionism can be divided into two dimensions: perfectionistic strivings and perfectionistic concerns [8]. The first dimension refers to an individual pursuit of perfection and the setting of high personal standards. Conversely, the perfectionistic concerns reflect the individual’s fear of making mistakes and negative reactions to defects. Some studies have investigated the relationship between perfectionism and attitudes toward doping among athletes [5, 6, 9–11], but no consensus has yet been reached. For example, a study by Bahrami et al. [6] found that perfectionistic strivings (personal standards) and perfectionistic concerns (concerns over mistakes) were positively correlated with attitudes toward doping. Bae et al. [11] found that concerns over mistake were positively correlated with attitudes toward doping. Another study showed that overall perfectionism was positively associated with attitudes toward doping [5]. However, Madigan et al. [10] observed a negative association between perfectionistic strivings and attitudes toward doping, and the study also found that perfectionistic concerns were not significantly related to attitudes toward doping. The reasons for the inconsistency are probably related to different instruments used to measure perfectionism and attitudes, different age and sporting levels of samples. Consequently, more studies are needed to further clarify the relationship between athletes’ perfectionism and doping attitudes.

In recent years, some studies have investigated attitudes toward doping based on different theories, of which self-determination theory (SDT) has received extensive attention [12–14]. SDT considers the individual’s motivation for engaging in an activity or task as a continuum of self-determination [15]. Intrinsic motivation is the motivation with the highest level of self-determination. That is to say, an individual engages in an activity or task for his or her inner pleasure. The other end of the continuum is amotivation, which means that an individual lacks intention and motivation. Extrinsic motivation falls between amotivation and intrinsic motivation. According to the self-determination of behavior, extrinsic motivation is further divided into four types of regulation: external regulation, introjected regulation, identified regulation, and integrated regulation. Among them, external regulation is a motivation with the lowest degree of self-determination, which primarily entails that individuals engage in certain activities or tasks to obtain rewards or to avoid punishment. Introjected regulation primarily means that individuals engage in certain activities or tasks to maintain self-esteem or avoid feelings of guilt; Identified regulation is a more autonomous motivation, which means that individuals understand and recognize the value of activities or tasks and accept them into the self. Integrated regulation is that individuals accept external goals and make them the core values and beliefs of the individuals concerned. Moreover, external regulation and introjected regulation were considered as controlled motivation, while intrinsic motivation, identified regulation and integrated regulation were considered as autonomous motivation [16].

Previous studies have shown that autonomous and controlled motivation can mediate the relationship between perfectionism and some variables among athletes, such as coping skills [17] and job burnout [18]. Therefore, the potential mediating role of autonomous and controlled motivation between athletes’ perfectionism and attitudes toward doping is worth investigating. In fact, studies have shown that autonomous and controlled motivation are correlated with past doping use and intentions for future use. Specifically, controlled motivation is positively associated with past doping use and intentions for future use [19, 20], while autonomous motivation is negatively associated with past doping use [19, 20] and intentions for future doping use [19]. Meanwhile, in the field of sports, the relationship between perfectionism and self-determined motivation was also investigated. Specifically, previous studies revealed that perfectionistic strivings had a positive relationship with autonomous motivation [18, 21], whereas perfectionistic concerns had a positive relationship with controlled motivation [18, 21, 22]. Therefore, based on the relationships among perfectionism, self-determined motivation and attitudes toward doping, it is possible that autonomous and controlled motivation may play a mediating role between perfectionism and athletes’ attitudes toward doping. However, no existing studies have investigated this possibility.

Taken together, this study, based on self-determination theory, was aimed to investigate the relationship among perfectionism, autonomous motivation and controlled motivation, and attitudes toward doping in athletes. The study proposed the following hypotheses (1) perfectionistic strivings negatively predicts attitudes toward doping, whereas perfectionistic concerns positively predicts attitudes toward doping; (2) autonomous motivation negatively predicts attitudes toward doping, whereas controlled motivation positively predicts attitudes toward doping; (3) autonomous motivation partially mediates the relationship between perfectionistic strivings and attitudes toward doping, while controlled motivation partially mediates the relationship between perfectionistic concerns and attitudes toward doping.

## Research methods

### Participants

In this study, a cluster sampling method was used to recruit participants from multiple professional sports teams in Shanghai and Jiangsu. Written informed consent was obtained from all participants. A total of 262 questionnaires were distributed and 243 valid questionnaires were returned. The rate of return was 92.7%. Among the participants, 128 were males (52.7%), and 115 were females (47.3%). Among them, 127 were first-level athletes (52.3%), 95 were national-level elite athletes (39.1%), and 21 were international-level elite athletes (8.6%). Among them, 41 were track and field athletes (16.9%), 46 were swimmers (18.9%), 38 were cyclists (15.6%), 25 were aerobics athletes (10.3%), 44 were basketball players (18.1%), 20 were tennis players (8.2%), and 29 were rowers (11.9%). The average age of the athletes was 20.5 years old (SD = 4.11).

### Measurement

#### Multidimensional perfectionism

Multidimensional perfectionism includes two dimensions: perfectionistic strivings and perfectionistic concerns. Multidimensional perfectionism was measured using four subscales encompassing a total of 25 items, namely “Striving for Perfection” (5 items, such as “I strive to be as perfect as possible” ), “Negative Reactions to Imperfection” (5 items, such as “I feel extremely stressed if everything does not go perfectly”), “Personal Standards” (7 items, such as “I have extremely high goals for myself in my sport”) and “Concern Over Mistakes” (8 items, such as “People will probably think less of me if I make mistakes in competition”). The “Personal Standards” and “Concern Over Mistakes” subscales were taken from the Sport Multidimensional Perfectionism Scale-2 (Sport-MPS-2) revised by Gotwals et al. [23]. The two subscales of “Striving for Perfection” and “Negative Reactions to Imperfection” were taken from the Multidimensional Inventory of Perfectionism in Sport (MIPS) revised by Madigan [24]. “Striving for Perfection” and “Personal Standards” represent “Perfectionistic strivings”. “Negative Reactions to Imperfection” and “Concern over Mistakes” represent “Perfectionistic concerns”. Using a Likert’s 5-point scoring scale, the scores ranged from “1” (completely disagree) to “5” (completely agree). The scale was translated into Chinese by three native Chinese speakers. After evaluating the consistency, the Chinese items were back-translated into English by three English translators. Two experts in the field evaluated the consistency between the English and Chinese versions. The Cronbach’s alpha coefficients of the four subscales employed in this study were between 0.82 and 0.88. The confirmatory factor analysis showed the two dimensions of perfectionistic strivings and perfectionistic concerns had good construct validity (χ^2^/df = 3.01, RMSEA = 0.06, NFI = 0.91, IFI = 0.94, CFI = 0.94; χ^2^/df = 3.41, RMSEA = 0.07, NFI = 0.90, IFI = 0.92, CFI = 0.92).

#### Autonomous and controlled motivation

Autonomous and controlled motivation were assessed by the Behavioral Regulation in Sport Questionnaire (BRSQ) [16]. A similar translation and back-translation procedure was used to create the Chinese version of BRSQ scale. The participants were asked to respond to the following stem “Below are some reasons why people participate in sport”. It contains a total of 24 items in six subscales: “Intrinsic Motivation” (4 items, such as “because I enjoy doing something to the best of my ability”), “Identified Regulation” (4 items, such as “because I value the benefits of my sport”), “Integrated Regulation” (4 items, such as “because it’s a part of who I am), “Introjected Regulation” (4 items, such as “because I would feel ashamed if I quit”), “External Regulation” (4 items, such as “because if I don’t other people will not be pleased with me”) and “Amotivation” (4 items, such as, “but I wonder what’s the point”). The items were scored using a 7-point Likert’s scale, ranging from “1″ (not at all true) to “7″ (very true). Autonomous motivation and controlled motivation were calculated with the following formulas: autonomous motivation: 2 × intrinsic motivation + identified regulation + integrated regulation; controlled motivation: 2 × introjected regulation + 2 × external regulation [12]. The Cronbach’s alpha coefficients of the subscales in this study ranged from 0.76 to 0.84. The confirmatory factor analysis showed that the questionnaire had good construct validity (χ^2^/df = 3.27, RMSEA = 0.07, NFI = 0.90, IFI = 0.93, CFI = 0.93).

#### Attitudes toward doping

The Performance Enhancement Attitude Scale (PEAS) adapted by Wang et al. [25] was used assessing attitudes toward doping. It comprises a total of 10 items, such as “Doping is necessary to be competitive”. The higher the score, the more the athletes tend to favor the use of doping. Using a 6-point Likert’s scoring method, the responses ranged from “1” (completely disagree) to “6” (completely agree). Cronbach’s alpha coefficient for the scale in this study was 0.89. The confirmatory factor analysis showed that the questionnaire had good construct validity (χ^2^/df = 3. 669, RMSEA = 0. 068, NFI = 0.907, IFI = 0.922, CFI = 0.921).

### Procedures and statistical analysis

Participants were surveyed in groups and informed consent was obtained from the sports team leaders and athletes before administration. The questionnaire was completed anonymously. Before the investigation, it was explained to the participants that the information and content they provide are only for scientific research and will be strictly confidential. Participants were asked to follow the guidelines carefully and respond independently. It took about 20 min for the participants to complete all the questionnaires, which were collected on the spot.

**Fig. 1:**
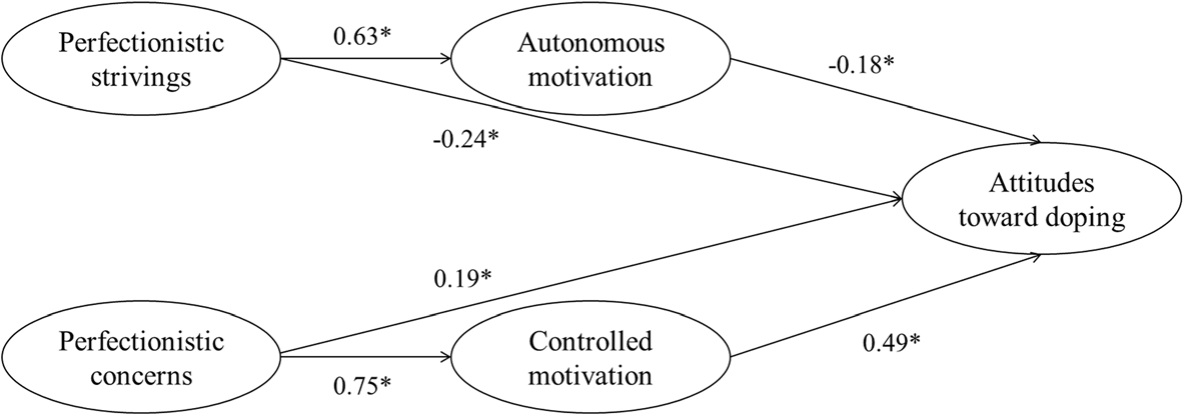
Structural equation modeling (* *P* < 0.05)

SPSS 20.0 (IBM Inc., Chicago, IL, USA) and AMOS 20.0 (IBM Inc., Chicago, IL, USA) packages were used for data analysis. First, Cronbach’s alpha coefficients and confirmatory factor analysis (CFA) were used to evaluate the reliability and construct validity of the scale. Pearson correlation coefficients were used to analyze relationships between perfectionism, autonomous motivation, controlled motivation and attitudes toward doping. Finally, structural equation modeling (SEM) was conducted to evaluate the mediating effects of autonomous and controlled motivation using maximum likelihood estimation. The average scores of the scales were used as manifest variables in the SEM analysis. Following the criteria from Imai et al. [26], Loeys et al. [27] and Rucker et al. [28], the potential mediating effects were evaluated. The statistical significance of the indirect effects was tested using a bootstrapping approach [29]. The standardized path coefficients were presented in Fig. 1 and Table 3.

## Results

### Descriptive statistics and correlation analysis

Table 1 presents the mean, standard deviation, and Cronbach’s alpha for each variable. The descriptive statistics indicate higher levels of the perfectionistic strivings among athletes. For the dimension of perfectionistic concerns, athletes showed moderate to low levels. This finding shows that young athletes have a higher tendency to pursue perfectionism. In addition, athletes demonstrated higher levels for all dimensions of autonomous motivation, and lower levels for each dimension of controlled motivation. These results indicate that young athletes have a more autonomous mode of motivational regulation. Athletes reported low levels of attitudes toward doping.

**Table 1:** Descriptive statistics and reliability coefficients

	Range	M	SD	Cronbach’s ɑ
Perfectionistic strivings				
Striving for perfection	1–5	4.26	0.55	0.82
Personal standards	1–5	4.00	0.66	0.83
Perfectionistic concerns				
Negative reactions to imperfection	1–5	3.14	0.87	0.88
Concern over mistakes	1–5	2.82	0.76	0.86
Autonomous motivation				
Intrinsic motivation	1–7	6.17	0.91	0.82
Identified regulation	1–7	6.00	0.92	0.80
Integrated regulation	1–7	5.93	0.93	0.84
Controlled motivation				
Introjected regulation	1–7	3.71	0.61	0.76
External regulation	1–7	2.98	0.81	0.79
Attitudes toward doping	1–6	1.49	0.76	0.89

Table 2 presents the correlation coefficients between the variables. Correlation analysis showed that perfectionistic strivings was significantly and negatively correlated with the attitudes toward doping (r = − 0.54, *P* < 0.01). There was a significant positive correlation between perfectionistic concerns and attitudes towards doping (r = 0.51, *P* < 0.01). Perfectionistic strivings was significantly and positively correlated with autonomous motivation (*r* = 0.52, *P* < 0.01), and there was a significant positive correlation between perfectionistic concerns and controlled motivation (r = 0.64, *P* < 0.01). There was a significant negative correlation between autonomous motivation and attitudes toward doping (*r* = − 0.43, *P* < 0.01), and there was a significant positive correlation between controlled motivation and attitudes toward doping (r = 0.59, *P* < 0.01).

**Table 2:** Correlation analysis for the study variables

Variable	1	2	3	4	5
1 Perfectionistic strivings	1				
2 Perfectionistic concerns	0.11	1			
3 Autonomous motivation	0.52**	−0.12	1		
4 Controlled motivation	−0.10	0.64**	−0.11	1	
5 Attitudes toward doping	−0.54**	0.51**	−0.43**	0.59* *	1

Note: * indicates *P* < 0.05, ** indicates *P* < 0.01

Meanwhile, a one-way Analysis of Variance (ANOVA) test revealed that there was no significant difference in attitudes toward doping among athletes participating in different sports (F = 0.067, *P* > 0.05). Post hoc analyses with pairwise comparison also showed that there was no significant difference in attitudes toward doping (all *P* > 0.05).

### Testing of mediation model

SEM was used to examine the mediating effects of autonomous motivation and controlled motivation on the influence of perfectionism on attitudes toward doping among young athletes. According to the mediating effect test procedure proposed by Wen et al. [30] two models were constructed in order to establish: (a) a direct effect model without mediating variables (autonomous motivation and controlled motivation) and (b) a mediation effect model containing mediating variables. The results show that the direct effect model fits well with the data (χ^2^/df = 4.21, RMSEA = 0.08, NFI = 0.95, IFI = 0.96, CFI = 0.96) and the standardized path coefficients for perfectionistic strivings–attitudes toward doping (β = − 0.42, *P* < 0.01) and perfectionist concerns–attitudes toward doping (β = 0.59, *P* < 0.01) were significant.

These results provide the necessary preconditions for the further construction of the mediation model. Autonomous motivation and controlled motivation were included as mediating variables to construct a mediating effect model. The results showed that the mediation model fits well (χ^2^/df = 3.61, RMSEA = 0.06, NFI = 0.90, IFI = 0.92, CFI = 0.92). The standardized path coefficients of the mediating effect model are shown in Fig. 1. The path coefficients for perfectionistic strivings–attitudes toward doping and perfectionistic concerns–attitudes toward doping were significant in both the direct effect model and the mediation effect model, but these were significantly lower in the mediation effect model, indicating that autonomous motivation partially mediates the relationship between perfectionistic strivings and attitudes toward doping. Controlled motivation partially mediates the relationship between perfectionistic concerns and attitudes toward doping.

To further confirm the mediating role of autonomous motivation and controlled motivation on the relationship between perfectionism and attitudes toward doping, this study also examined the full mediation model. The results showed that although the full mediation model also fit well (χ^2^/df = 3.66, RMSEA = 0.07, NFI = 0.89, IFI = 0.91, CFI = 0.91), compared with the partial mediation effects model, the χ^2^ value increased significantly (∆χ^2^ = 13.46, ∆df = 1, *P* < 0.01), and the fit indices of χ^2^/df and NFI were not as favorable, indicating the path for perfectionistic strivings–attitudes toward doping and perfectionistic concerns–attitudes toward doping should not be removed. These results further confirm that autonomous motivation and controlled motivation play a partial mediating role in the relationship between perfectionism and attitudes toward doping.

According to the results of path analyses (Fig. 1 and Table 3), perfectionistic strivings had negative and direct effects on attitudes toward doping (− 0.24), whereas perfectionistic concerns had positive and direct effects on attitudes toward doping (0.19). Perfectionistic strivings had positive and direct effects on autonomous motivation (0.63), and perfectionistic concerns had positive and direct effects on controlled motivation (0.75). Autonomous motivation had negative and direct effects on attitudes toward doping (− 0.18), whereas controlled motivation had positive and direct effects on attitudes toward doping (0.49). Perfectionistic strivings had an indirect effects on attitudes toward doping through autonomous motivation (− 0.11), and perfectionistic concerns had an indirect effect on attitudes toward doping through controlled motivation (0.37).

**Table 3:**
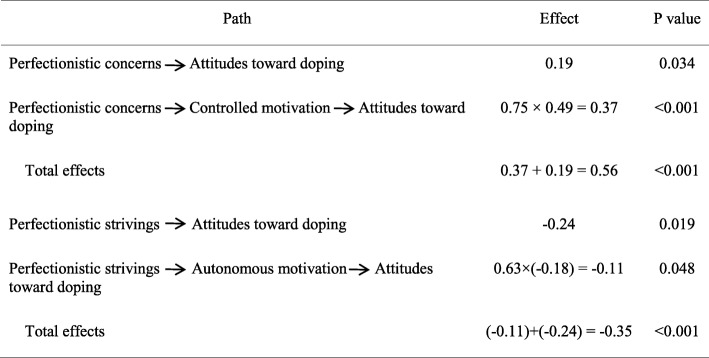
Results of path analysis

Note: the standardized path coefficients were presented

## Discussion

This study aimed to examine the relationship between multidimensional perfectionism and attitudes toward doping among young athletes, as well as the mediating role of autonomous and controlled motivation between multidimensional perfectionism and attitudes toward doping.

In terms of perfectionistic strivings, this study found that perfectionistic strivings can negatively predict athletes’ attitudes toward doping. The findings largely supported previous studies [9, 10]. In terms of perfectionistic concerns, this study found that perfectionistic concerns were a significant and positive predictor for attitudes toward doping among athletes. Bahrami et al. [6] and Bae et al. [11] found a positive correlation between perfectionistic concerns and attitudes towards doping, while Madigan et al. [10] found no significant association between perfectionistic concerns and attitudes toward doping. Stoeber et al. [8] pointed out that perfectionistic strivings refers to the tendency to set high personal standards or the internal driving force for excellence, which is an adaptive personality tendency. In contrast, perfectionistic concerns is a form of non-adaptive perfectionism, which refers to constantly worrying about mistakes, fear of failure, severe self-criticism, self-worth depending on performance, and negative emotional reactions to differences between self-expectations and actual performance [8, 31]. The two dimensions of perfectionism have distinct relationships with positive and negative psychological outcomes. Studies have shown that perfectionistic strivings are positively correlated with self-confidence in competition [32], positive emotional reactions to sports success [33], and mastery and performance-approach goal orientation [34]. However, perfectionistic concerns are positively related to fear of failure [33] and competitive anxiety [33, 35]. Therefore, individuals who hold perfectionistic strivings seek success, set higher personal standards, and their goal is to do their utmost to realize their potential, and subsequently obtain satisfaction and self-affirmation, while deception (such as the use of doping) will undermine the value process of achieving success. Those with perfectionistic concerns are afraid of failure, fear being criticized by others. In order to avoid poor performance and its negative results, as well as people’s negative evaluation of their abilities, it is more likely to accept cheating behaviors (such as doping).

This study showed that autonomous motivation is negatively correlated to attitudes toward doping, and controlled motivation is positively correlated to attitudes towards doping. This is consistent with the results of previous studies [19, 20]. Therefore, athletes’ autonomous and control motivation affect their attitudes toward doping, and different forms of motivation have distinct effects on their attitudes toward doping. According to the self-determination theory, the behavior of athletes with a tendency toward autonomous motivation is primarily derived from the real self, and they strive to satisfy their psychological needs for competence, autonomy, and relationships. Enjoyment arises from efforts to improve and perfect performance by appropriate means, engaging in activities that meet goals and values, and establishing relationships with others [36], rather than from winning at all costs (such as doping). The use of doping by athletes with a tendency toward autonomous motivation will violate these psychological needs because they would be engaging in acts that are contrary to their goals and values, gaining false abilities, and interfering with other athletes by fraudulent and unfair means to gain an advantage over their opponents [36]. It is suggested that psychological need satisfaction and self-determined motivation are significantly associated with young athletes’ moral attitudes, and athletes’ attitudes toward antisocial behaviors predict subsequent rule violations [37]. Therefore, athletes with a tendency toward autonomous motivation are less likely to adopt antisocial behaviors such as doping use. In contrast, athletes with a tendency toward controlled motivation primarily act for self-improvement and to obtain fame and extrinsic rewards. These athletes pay more attention to the outcome of the competition and “winning” so that they can achieve their goals for self-improvement, fame, and reward, thereby satisfying their self-esteem. Athletes who overemphasize the outcome of the match and winning the game may be tempted to do anything to ensure a victory [38, 39], and thus have a positive attitude toward the use of doping.

This study further showed that autonomous and controlled motivation play a mediating role of the relationship between multidimensional perfectionism and attitudes toward doping. Controlled motivation is a partial mediator between perfectionistic concerns and attitudes toward doping. Perfectionistic concerns involves motives (introjected regulation and external regulation) that are only partially self-internalized. Perfectionistic concerns are more likely to lead to internal psychological stress because athletes who are concerned about mistakes and negative reactions to imperfections are more likely to associate these concerns with self-worth. If so, these athletes are more likely to form (or maintain) controlled motivation. According to self-determination theory, athletes dominated by introjected regulation and/or external pressures, such as guilt or shame, fear of punishment, etc., are more likely to be influenced by “unscrupulous” ideas [40], and are more likely to violate the spirit of the game by employing deception, including the use of doping.

Autonomous motivation plays a partial mediation role between perfectionistic strivings and athletes’ attitudes toward doping. Perfectionistic strivings may encourage individuals to integrate sports participation to a greater degree as part of the self. Unlike those with perfectionistic concerns, individuals with perfectionistic strivings have more adaptive reasons for athletic participation. Athletes who set high personal standards and pursue perfection are more likely to find that the sport is fun, enjoyable, valuable, or has great personal significance. In a meaningful way, these athletes are more likely to form (or maintain) autonomous motivation. According to self-determination theory, athletes dominated by intrinsic motivation and/or identified regulation, such as pleasure, value of participation in sports, etc., are more likely to adopt prosocial and ethical behaviors, such as helping others, obeying the rules of the game, avoid doping.

## Conclusion

The study finds that self-determined motivations partially mediate the relationship between perfectionism and athletes’ attitudes toward doping. Controlled motivation plays a partial mediation role between perfectionistic concerns and attitudes toward doping. Autonomous motivation partially mediates the relationship between perfectionistic strivings and attitudes toward doping. Therefore, fostering athletes’ perfectionistic strivings and autonomous motivation and reducing perfectionistic concerns and controlled motivation may be an effective education strategy for lowering attitudes toward doping among athletes.

## Data Availability

The data and material are available upon reasonable request.

## Abbreviations

ANOVA: Analysis of Variance
BRSQ: Behavioral Regulation in Sport Questionnaire
CFA: Confirmatory Factor Analysis
CFI: Comparative Fit Index
IFI: Incremental Fit Index
NFI: Normed Fit Index
RMSEA: Root Mean Square Error of Approximation
SD: Standard Deviation
SDT: Self-determination Theory
SEM: Structural Equation Modeling
